# Capivasertib‐Associated Diabetic Ketoacidosis With Concurrent Myocardial Injury in a Patient With Pre‐Existing Coronary Artery Disease

**DOI:** 10.1155/crie/6940757

**Published:** 2026-10-11

**Authors:** Rabih A. Noun, Ossama Sajeh Maadarani, Zouheir I. Bitar

**Affiliations:** ^1^ Cardiology Specialist Internal Medicine Department Ahmadi Hospital Ahmadi Kuwait; ^2^ Consultant Cardiologist Internal Medicine Ahmadi Hospital Kuwait Oil Company Ahmadi Kuwait kockw.com; ^3^ Consultant in Critical Care Medicine Internal Medicine Department Ahmadi Hospital Ahmadi Kuwait

**Keywords:** capivasertib, ischemic heart disease, ketoacidosis

## Abstract

Capivasertib is a serine/threonine kinase AKT (AKT1–3) inhibitor, disrupting phosphorylation of AKT substrates. When combined with fulvestrant, it is approved for hormone receptor‐positive advanced breast cancer. Capivasertib therapy frequently causes hyperglycemia, including diabetic ketoacidosis (DKA). Ensure glycemic control before initiation and maintain ongoing blood glucose monitoring to reduce the risk of this potentially fatal complication. We present a case of metastatic breast cancer with initially controlled blood glucose who developed ketoacidosis and myocardial infarction during capivasertib therapy.

**Highlights**

• Rigorous blood glucose monitoring and prompt glycemic management are critical to prevent severe ketoacidosis in patients with diabetes.
• Capivasertib may be associated with ischemic cardiac events; differentiating the causality between DKA and drug effect remains complex.

## 1. Introduction

The antineoplastic agent capivasertib is a serine/threonine kinase AKT (AKT1‐3) inhibitor used to treat locally advanced or metastatic breast cancer. The majority of advanced breast cancers, almost 70%, express the hormone receptors estrogen or progesterone and do not have human epidermal growth factor receptor 2 (HER2) overexpression [1]. In patients with advanced breast cancer, the aromatase inhibitor (endocrine therapy), often first‐line therapy and sometimes combined with a cyclin‐dependent kinase 4 and 6 (CDK4/6) inhibitor, has been shown to notably improve prognosis compared with aromatase inhibitor therapy alone [2].

Hyperglycemia is a common problem in patients on regular capivasertib therapy; ~16% experience it. In rare cases, this can become severe, with life‐threatening ketoacidosis occurring in only 0.3% of cases, which represents very few patients, and only a few instances have been reported in the literature [3]. The SOLAR‐1 trial observed a high discontinuation rate during alpelisib therapy due to hyperglycemia (any‐grade events in 64% of patients and grade ≥3 in 37%) [4].

We report severe diabetic ketoacidosis (DKA), induced by the antineoplastic agent capivasertib in a patient with advanced breast cancer and inadequately controlled diabetes during therapy, complicated by myocardial infarction that required coronary angiography.

## 2. Case Description

A 67‐year‐old female with type 2 diabetes mellitus (T2DM), well controlled, managed with metformin1 g twice daily, and metastatic breast carcinoma involving the pulmonary and lymphatic systems, underwent a modified radical left mastectomy in 2001, followed by chemotherapy and radiotherapy (Figure 1). Seven months before her emergency admission, she commenced endocrine therapy with capivasertib and fulvestrant. At initiation, her baseline HbA1c was 5.6%. Four weeks after starting the new therapy, her glucose control deteriorated, which her physician addressed with vildagliptin; according to the patient, euglycemia was restored. Over the next 6 months, she continued capivasertib and fulvestrant, but fasting blood sugar was reported to be elevated between 10 and 12 mmol/L, and the patient did not seek medical advice. Seven months after initiation, she presented to the emergency department with a 2‐day history of generalized fatigue, recurrent emesis, retrosternal burning pain radiating to the oropharynx, and diarrhea. Notably, she also has coronary artery disease (CAD), a history of angina, and prior coronary angiography at another center.

**Figure 1:**
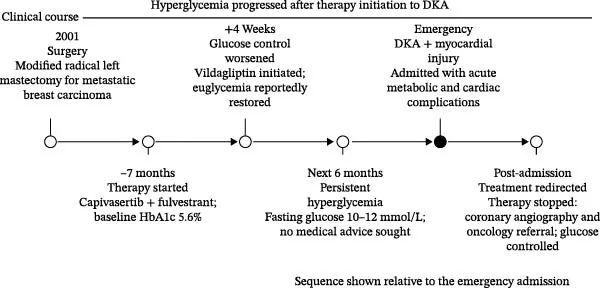
Patient timeline.

The patient was clinically dehydrated. Her blood pressure was 144/70 mmHg. Her heart rate was 112 beats per minute. Her respiratory rate was 25 breaths per minute. Table 1 summarizes the initial laboratory results. Blood glucose was 24 mmol/L (reference range: 4–6.2 mmol/L). Beta‐hydroxybutyrate was 6.5 mmol/L (reference range: 0.05–0.27 mmol/L). Serum bicarbonate was 13.7 mmol/L (reference range: 22–28 mmol/L). Venous blood gas pH was 7.03, consistent with metabolic acidosis. Serum sodium, corrected for hyperglycemia, was 132 mmol/L (reference range: 135–145 mmol/L). Potassium was 5.1 mmol/L (reference range: 3.5–5.1 mmol/L). Renal function was compromised. Serum creatinine was 234 μmol/L (reference range: 60–120 μmol/L). These findings indicated high anion gap metabolic acidosis due to mixed DKA and acute kidney injury. Urinalysis revealed no infection. C‐reactive protein was normal (5 mg/L). Toxicology screens for methanol, ethylene glycol, and diethylene glycol were negative. There was no osmolar gap. Electrocardiography showed isolated ST elevation in V2 consistent with a possible non‐ST‐elevation acute myocardial infarction (Figure 2). Serial troponin concentrations had a significant rising pattern and are displayed in Table 1.

**Figure 2:**
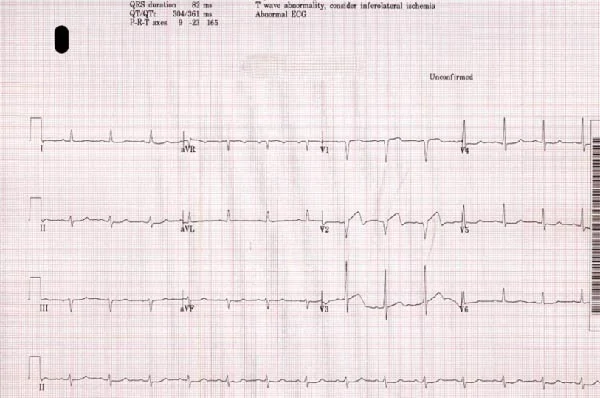
ECG shows ST elevation in V2.

**Table 1:** Laboratory results.

Values (reference range)	On admission	12 h	24 h
White blood count × 10^9^/L	7.3	11.3	—
Hemoglobin, g/dL	13.8	12.5	—
Platelet count × 10^9^/L	314	287	—
Sodium level, mmol L	132	142	141
Potassium, mmol/L	5.1	4.2	3.7
Creatinine, umol/L	234	167	96
Blood urea nitrogen, mmol/L	12.4	8.0	5
Chloride level, mmol/L	93	109	110
HCO3, mmol/L	13.7	15.2	20
Anion gap	25.3	16	12
Total bilirubin, umol/L	6	—	—
Beta‐hydroxybutyrate, mmol/L (0.05–0.27 mmol/L)	6.5	—	0.6
HBA1c %	10.4	—	—
C peptide, ng/ml (0.8–3.9)	—	3.2	—
Magnesium, mmol/L	0.7	0.9	—
Phosphorus, mmol/L	1.1	0.78	—
Corrected calcium, mmol/L	—	2.13	—
Troponin, pg/ml	34.4	118.6	560
Amylase, u/l	—	27	—
Lipase	—	50 u/L	—
Albumin, g/L	43	—	31

The patient was started on a DKA management protocol, including continuous intravenous insulin and intravenous fluids. The patient was started on short‐acting insulin via continuous IV infusion using an infusion pump at a rate of 0.1 U/kg/h. A mix of 24 units of regular insulin in 60 mL of isotonic sodium chloride solution is usually infused at a rate of 15 mL/h (6 U/h) until the blood glucose level drops to around 10 mmol/L; the rate of infusion then decreases to 5–7.5 mL/h (2–3 U/h) until the ketoacidosis state is resolved. The glucose drop rate is 5 mmol/L/h. We did not allow the blood glucose level to fall below 11 mmol/L during the first 5 h of treatment.

We initially corrected fluid loss with lactated Ringer’s solution. We administered 1 L during the first hour. We then administered another 1 L during the second hour. Then we administered 1 L over the following 2 h.

We closely monitored the renal function, which ultimately returned to normal. The intravenous insulin infusion continued until both ketonemia and the anion gap were resolved. The patient was then transitioned to a subcutaneous basal‐bolus insulin regimen.

The patient received dual antiplatelet therapy with acetylsalicylic acid and clopidogrel, and was also administered a heparin infusion for anticoagulation. Coronary angiography demonstrated a chronic total occlusion of the left anterior descending coronary artery, which was managed conservatively with medical therapy.

Capivasertib was discontinued due to a clear, time‐dependent onset of severe hyperglycemia; the patient transitioned from a normal baseline HbA1c of 5.6% to 10.4% ~4 weeks after initiating the drug and DKA 7 months later, with subsequent glycemic improvement following its discontinuation. The patient was referred to medical oncology for alternative cancer treatment options.

## 3. Discussion

Oncologic checkpoint inhibitors can induce DKA via maladaptation or autoimmune destruction of pancreatic beta cells [5], and oncologic targeted therapies, such as alpelisib, do so by inhibiting glucose transporter 4 and glycogen synthesis pathways [6].

Capivasertib achieves anti‐tumor activity by inhibiting serine/threonine kinases (AKT), which also mediate insulin signaling by promoting glucose uptake in tissues and hepatic glycogen storage [7]. This inhibition significantly disrupts insulin sensitivity, directly leading to insulin resistance and hyperglycemia. By impairing downstream insulin signaling—primarily by inhibiting AKT phosphorylation—capivasertib reduces glucose transporter translocation to the cell surface and suppresses glycogen synthesis. These effects increase hepatic gluconeogenesis, decrease glucose uptake in muscle and adipose tissue, and result in hyperglycemia [8, 9].

Hyperglycemia in controlled diabetes mellitus and an increase in fasting glucose from baseline in nondiabetic patients occurred in more than one‐third of capivasertib‐treated patients, with a median time to first occurrence of 15 days [10]. Severe DKA had been reported in capivasertib‐treated diabetic and nondiabetic patients [8]. Treating physicians should monitor blood sugar in nondiabetic patients, given the expanding use of AKT inhibitors in oncology [11]. Clinicians should watch for possible hyperglycemia and DKA in patients without a history of diabetes [11]. Previous literature has primarily documented capivasertib‐induced DKA in patients without a prior history of diabetes. The present case report demonstrates the development of severe DKA in a patient with previously well‐controlled type 2 diabetes mellitus (T2DM), accompanied by concurrent acute myocardial injury.

According to guidelines and expert opinion, educate patients that capivasertib may cause hyperglycemia, with a median time to onset of 15 days (range, 1–51 days). Consider prophylactic metformin for patients at increased risk (e.g., HbA1c ≥ 5.7%, prediabetes, BMI ≥ 30 kg/m2) or with an elevated baseline fasting blood sugar measured 1 week before initiating capivasertib [10, 12]. Monitor or self‐monitor fasting blood sugar levels on day 3 or 4 of the dosing week during weeks 1, 2, 4, 6, and 8, then monthly while on treatment and as clinically indicated. Check HbA1c every 3 months while on treatment and as clinically indicated. Patients with a history of well‐controlled type 2 diabetes may require intensified antihyperglycemic therapy and closer fasting blood sugar monitoring. Consider consultation with a health care provider experienced in managing hyperglycemia, with fasting blood sugar monitoring at least twice weekly on both capivasertib dosing and nondosing days, until fasting glucose returns to baseline. During antidiabetic treatment, monitor fasting blood sugar weekly for 2 months, then biweekly, or as clinically indicated [10, 12]. *If hyperglycemia cannot be controlled with proper use of antihyperglycemic drugs*, *dose modifications of Capivasertib may be sought* [12].

The present case report describes a diagnosis of non‐ST‐elevation myocardial infarction with DKA. In case reports of patients with DKA, normal coronary angiography, and pH below 7, elevated troponin is postulated to result from severe acidemia, increasing intracellular calcium [13]. Severe acidosis disrupts the interaction between calcium and contractile proteins, causing myocardial stunning. Ultimately, this combination of proteolysis and myocardial stunning increases serum marker levels [14]. The relationship between acute coronary syndrome, DKA, and elevated troponin is complex.

Severe DKA can precipitate myocardial ischemia through dehydration and hypovolemia, leading to hypoperfusion of cardiac tissue. Moreover, metabolic acidosis with ketosis impairs myocardial cells’ sensitivity to calcium, thereby promoting vasoconstriction [15]. These factors contribute to a significant oxygen supply‐demand mismatch (Type 2 myocardial infarction) [15].

In acute myocardial ischemia, counter‐regulatory stress hormones (epinephrine, cortisol, and glucagon) increase, along with insulin resistance and accelerated lipolysis, precipitating hyperglycemia and worsening DKA in susceptible individuals [16]. Pre‐existing CAD plays a critical underlying role in this association, as subclinical or established coronary atherosclerosis significantly lowers the ischemic threshold, enabling metabolic and hemodynamic stress to reveal clinically overt myocardial injury. Elevated troponin levels in DKA may therefore indicate underlying coronary pathology unmasked by metabolic stress, or direct noncoronary myocyte toxicity resulting from severe acidosis, insulin deficiency, and elevated circulating free fatty acids [17]. Thus, troponin elevation in a diabetic patient should be regarded as indicative of a coronary abnormality until proven otherwise, particularly since delayed troponin kinetics do not permit reliable differentiation between coronary and noncoronary causes [16].

An additional diagnostic dilemma arises from the concurrent use of capivasertib, raising suspicion of drug‐induced cardiac ischemia. Beyond its well‐established metabolic complications, capivasertib has not shown a clear, direct mechanistic association with vascular endothelial toxicity or coronary vasospasm. However, ischemic cardiac events have been documented during the treatment. In the phase 3 CAPItello‐291 trial, serious adverse events occurred in 57 patients (16.1%) receiving capivasertib–fulvestrant vs. 28 (8.0%) receiving placebo–fulvestrant; death due to adverse events occurred in 4 patients (1.1%) in the capivasertib group, including one death from acute myocardial infarction (though none were investigator‐adjudicated as drug‐related) [18]. Whether capivasertib directly increases ischemic cardiac risk remains unproven.

The limitation of the report is that this *is a single case report; the temporal association between capivasertib administration and the onset of DKA and myocardial infarction cannot definitively establish direct drug causality*.

## 4. Conclusion

This case report highlights the need for glucose monitoring in all patients receiving PI3K/AKT pathway inhibitors, regardless of diabetes status, and for early intervention. Ischemic cardiac events are possible, but it is difficult to confirm whether they are related to DKA or drug‐induced. Patients with DKA have increased troponin levels, which may reflect either pre‐existing coronary heart disease or normal coronary arteries.

## Author Contributions

Zouheir I. Bitar wrote the article. Rabih A. Noun shared in writing the case presentation. Ossama Sajeh Maadarani shared in the discussion on collecting the data and revising the manuscript.

## Funding

The authors received no financial support for the research, authorship, and/or publication of this article.

## Disclosure

The authors performed the research as part of their employment with Kuwait Oil Company.

## Consent

Written informed consent for the publication of this case was obtained from the patient.

## Conflicts of Interest

The authors declare no conflicts of interest.

## Data Availability

The data that support the findings of this study are available upon request from the corresponding author. The data are not publicly available due to privacy or ethical restrictions.

## References

[1] Howlader N. , Altekruse S. F. , and Li C. I. , et al.US Incidence of Breast Cancer Subtypes Defined by Joint Hormone Receptor and HER2 Status, JNCI: Journal of the National Cancer Institute. (2014) 106, no. 5, 10.1093/jnci/dju055, dju055. PMC4580552 24777111

[2] Gennari A. , André F. , and Barrios C. H. , et al.ESMO Clinical Practice Guideline for the Diagnosis, Staging, and Treatment of Patients With Metastatic Breast Cancer, Annals of Oncology. (2021) 32, no. 12, 1475–1495, 10.1016/j.annonc.2021.09.019. 34678411

[3] Chu A. , Hakami-Majd N. , and Whall J. O. , Acute Hyperglycemic Hyperosmolar Syndrome Associated With Capivasertib, a New Oral Targeted Therapy for Advanced Breast Cancer, American Journal of Emergency Medicine. (2025) 88, 273.e5–273.e7, 10.1016/j.ajem.2024.12.006. 39690017

[4] André F. , Ciruelos E. , and Rubovszky G. , et al.Alpelisib for PIK3CA-Mutated, Hormone Receptor–Positive Advanced Breast Cancer, New England Journal of Medicine. (2019) 380, no. 20, 1929–1940, 10.1056/NEJMoa1813904. 31091374

[5] Nagy S. , Demory Beckler M. , Hussein A. , and Kesselman M. M. , The Development of Diabetes and Diabetic Ketoacidosis Following Immunotherapy Treatment: A Systematic Review of Case Reports, Cureus. (2024) 16, no. 4, 10.7759/cureus.57894, e57894. PMC11007471 38606021

[6] Fugere T. , Roy A. M. , and Makhoul I. , Alpelisib-Induced Diabetic Ketoacidosis in a Nondiabetic Patient, Cureus. (2021) 13, no. 11, 10.7759/cureus.19295, e19295. PMC8648282 34900474

[7] Miao R. , Fang X. , Wei J. , Wu H. , Wang X. , and Tian J. , Akt: A Potential Drug Target for Metabolic Syndrome, Frontiers in Physiology. (2022) 13, 10.3389/fphys.2022.822333, 822333. PMC8940245 35330934

[8] Darapu H. and McKnight M. , Capivasertib-Induced Refractory Hyperglycemia in a Nondiabetic Patient With Metastatic Breast Cancer, AACE Endocrinology and Diabetes. (2025) 13, no. 2. 10.1016/j.aed.2025.10.011 PMC13043469 41938274

[9] Miglietta F. , Razeti M. G. , and Caltavituro A. , et al.The Evolving Landscape of Hormone Receptor-Positive/HER2-Negative Metastatic Breast Cancer (EVOLVE): An Italian Delphi Consensus Report, Critical Reviews in Oncology/Hematology. (2025) 213, 10.1016/j.critrevonc.2025.104793, 104793. 40484157

[10] Iyengar N. M. , O’Shaughnessy J. A. , and Moore H. N. , et al.Optimizing Clinical Monitoring and Management Guidelines for Capivasertib in HR-Positive/HER2-Negative Advanced Breast Cancer: Expert Opinion, npj Breast Cancer. (2026) 12, no. 1, 10.1038/s41523-025-00864-2, 16. PMC12820357 41345397

[11] Habeb B. , Valdes O. , Khair S. , Sidhu G. , and Fowler M. , Capivasertib-Induced Diabetic Ketoacidosis in a Non-Diabetic Patient With Metastatic Prostate Cancer With Bone Involvement: A Case Report of a Rare but Serious Metabolic Complication, Cureus. (2025) 17, no. 3, 10.7759/cureus.81513, e81513. PMC12043024 40308416

[12] Moore H. N. , Goncalves M. D. , and Johnston A. M. , et al.Effective Strategies for the Prevention and Mitigation of Phosphatidylinositol-3-Kinase Inhibitor-Associated Hyperglycemia: Optimizing Patient Care, Clinical Breast Cancer. (2025) 25, no. 1, 1–11. 10.1016/j.clbc.2024.09.017 39462728

[13] Moller N. , Foss A. C. , and Gravholt C. H. , et al.Myocardial Injury With Biomarker Elevation in Diabetic Ketoacidosis, Journal of Diabetes and its Complications. (2005) 19, no. 6, 361–363, 10.1016/j.jdiacomp.2005.04.003. 16260354

[14] Moin K. , Amini Z. , and Umar R. , Concurrent Diabetic Ketoacidosis and Non-ST-Elevation Myocardial Infarction: A Complex Cardiometabolic Emergency, Cureus. (2026) 18, no. 1, e101274. 10.7759/cureus.101274 PMC12889189 41674770

[15] López D. E. S. , Jiménez V. E. D. , Hernández N. C. C. , Vázquez G. N. , Robles M. E. G. , and Carrillo M. A. R. , Acute Myocardial Infarction in the Context of Diabetic Ketoacidosis: Pathophysiological Interactions, Clinical Manifestations, and Therapeutic Considerations, International Journal of Medical Science and Clinical Research Studies. (2024) 04, no. 10, 10.47191/ijmscrs/v4-i10-39.

[16] Kaefer K. , Botta I. , and Mugisha A. , et al.Acute Coronary Syndrome and Diabetic Ketoacidosis: The Chicken or the Egg?, Annals of Translational Medicine. (2019) 7, no. 16, 397. 10.21037/atm.2019.07.38 PMC6736820 31555711

[17] Jeremias A. and Gibson C. M. , Narrative Review: Alternative Causes for Elevated Cardiac Troponin Levels When Acute Coronary Syndromes Are Excluded, Annals of Internal Medicine. (2005) 142, no. 9, 786–791, 10.7326/0003-4819-142-9-200505030-00015. 15867411

[18] Turner N. C. , Oliveira M. , and Howell S. J. , et al.CAPItello-291 Study Group, New England Journal of Medicine. (2023) 388, no. 22, 2058–2070, 10.1056/NEJMoa2214131. PMC11335038 37256976

